# Comment on Rao et al. The Oncological Outcome of Postoperative Radiotherapy in Patients with Node-Negative Early-Stage (T1/T2/N0) Oral Squamous Cell Carcinoma and Perineural Invasion: A Meta-Analysis. *Cancers* 2025, *17*, 862

**DOI:** 10.3390/cancers18101520

**Published:** 2026-05-09

**Authors:** Ganesh Datta Borewad, Bhavya Kanakarajulu, Zayba Noor, Arun Krishna, Shalini Thakur, Anand Subash, Vishal U. S. Rao

**Affiliations:** Department of Head & Neck Surgical Oncology, HCG Cancer Centre, Bangalore 560027, India

We read with great interest the recent meta-analysis by Rao et al. evaluating the oncological benefit of postoperative radiotherapy (PORT) in patients with early-stage (T1/T2N0) oral squamous cell carcinoma (OSCC) with perineural invasion (PNI) [1]. The authors are to be commended for addressing an important and clinically relevant question in an area where evidence remains limited and decision-making is often nuanced. However, we wish to highlight several methodological and interpretative issues that may influence the validity and clinical applicability of the findings.

Lack of Detailed Characterization of PNI:

Only two of the included studies explicitly defined criteria for PNI. Critical prognostic variables—such as focal versus multifocal PNI, nerve caliber, and extent of involvement—were not consistently reported. Prior studies have demonstrated that multifocal PNI is associated with significantly worse oncological outcomes compared with unifocal PNI, even in early-stage OSCC [2,3]. The absence of such stratification limits risk adjustment and may lead to an overestimation of the benefit of PORT in biologically low-risk disease.

Inclusion of the Study by Holcomb et al.:

The study by Holcomb et al. included patients with pN0–N1 disease and incorporated lymphovascular invasion (LVI) as a variable [4]. This appears to conflict with the stated inclusion criteria of pN0 disease with PNI as the sole adverse feature. Inclusion of patients with nodal involvement or additional high-risk pathological features may confound survival outcomes attributed solely to PORT.

Ambiguity in the Study by Singareddy et al.:

The original study by Singareddy et al. focused exclusively on carcinoma of the oral tongue, whereas Table 1 of the meta-analysis categorizes the site as “oral cavity” [5]. Given the known prognostic and biological differences between oral tongue and other oral subsites, this generalization may be misleading. Furthermore, this was the only study to provide detailed radiotherapy protocol information, underscoring heterogeneity in treatment reporting across the included studies.

Inclusion of Chemotherapy in the Study by Cheng et al.:

The study by Cheng et al. included patients who received postoperative chemoradiotherapy rather than PORT alone [6]. As the meta-analysis aimed to isolate the effect of adjuvant radiotherapy in T1–T2N0 disease, inclusion of systemic therapy introduces a significant confounder and may inflate the perceived benefit of PORT.

Criteria Conflict in the Study by Chen et al.:

Patients in the study by Chen et al. were included based on the presence of PNI and/or LVI [7]. Since LVI is an independent adverse prognostic factor, combining these variables conflicts with the meta-analysis premise of evaluating PNI as the sole adverse feature, potentially skewing oncological outcome estimates.

Study by Nair et al. and Stage Misclassification:

The study by Nair et al. administered adjuvant therapy to patients with depth of invasion ≥10 mm and poor tumor differentiation—features that are more consistent with T3 disease under contemporary staging systems rather than early-stage T1–T2 tumors [8]. Additionally, the definition of disease-free survival differed between the studies by Nair et al. and Cheng et al., further affecting outcome uniformity.

Numerical Discrepancy in Cohort Size:

A discrepancy exists in the reported cohort size: the Abstract cites 522 patients (281 PORT, 241 no-PORT), whereas the Results section and Table 1 list 248 patients in the no-PORT group. Clarification of this inconsistency is essential to maintain data integrity and reader confidence.

Conclusion:

While this meta-analysis addresses a highly pertinent clinical question, the inclusion of studies with heterogeneous eligibility criteria and additional confounding prognostic factors warrants cautious interpretation. A refined analysis excluding such studies—or providing explicit justification for their inclusion—would further strengthen the robustness and clinical applicability of the conclusions, particularly in guiding adjuvant treatment decisions for early-stage OSCC with isolated PNI.

## Data Availability

No new datasets were generated or analyzed during the current study.
